# Using Simulation to Teach Methods for Improving Patient Literacy about Medicines

**DOI:** 10.3390/pharmacy8040192

**Published:** 2020-10-18

**Authors:** Vivienne Mak, Anisha Kaur Sandhu, Sunanthiny Krishnan

**Affiliations:** 1 Faculty of Pharmacy and Pharmaceutical Sciences, Monash University, Parkville, VIC 3052, Australia; 2 School of Pharmacy, Monash University Malaysia, Bandar Sunway, Selangor 47500, Malaysia; anisha.kaur@monash.edu (A.K.S.); Sunanthiny.S.Krishnan@monash.edu (S.K.)

**Keywords:** pharmacy education, health literacy, simulation training

## Abstract

Pharmacists have a role in educating patients on the self-management of their medications, using accurate medicines lists. Thus, pharmacy students need to be adequately trained and assessed in health-literacy skills to be competent for future patient-education consultations using medicines lists. Performance-based assessments using patient simulation are often utilized to examine students’ competence in clinical knowledge and communication skills. Due to COVID-19, education systems changed to remote online delivery utilizing video conferencing platforms (i.e., Zoom^TM^), which proved challenging for performance-based assessments. These challenges include difficulty in observing non-verbal cues over Zoom^TM^ and not having adequate internet access. Adaptations to reduce reliance on internet access were made where students submitted a video-recording task wherein they educated a simulated patient on a medicines list, under lockdown restrictions during the pandemic. A total of 304 submissions were received where students performed the role-play with a simulated patient, such as their family members, housemates or peers either at home in person or via Zoom^TM^. Although it was not an original goal of the task, the collaborative effort between pharmacy instructors, students and the public helped increase awareness of medicines lists through this task.

## 1. Introduction

Access to healthcare has changed drastically during the coronavirus disease 2019 (COVID-19) pandemic, as governments worldwide observe measures to contain the virus. These include different levels of restriction to movement, such as partial or nationwide lockdowns, self-isolation and social distancing [1,2,3]. As a result, the public has had to adapt health behaviors rapidly and come to rely on the internet and social media for support, health information and updates on the virus [1,4,5,6,7]. This reinforces the importance of health literacy, as the public is required to navigate through a daily deluge of rumor, misinformation and false news, in order to seek, understand and apply evidence-based health information in a safe manner [1,4,5,8]. Patients with chronic diseases are also deemed to be at a higher risk of threat from COVID-19 [3]. In addition to the concerning impacts of stress and anxiety brought on by the pandemic, they also have to take on a greater role in self-management of their medications during this time [3]. Hence, the COVID-19 pandemic provides a timely opportunity to better educate patients on using their medications safely, for improving health literacy and ensuring the quality use of medicines [9].

Pharmacists worldwide have been essential on the medical frontline, carrying out impactful interventions by ensuring consistent medication supply and providing medication-adherence support in the form of patient education and counselling to manage chronic conditions, optimize medication use and prevent medication errors [3,9,10,11,12,13,14,15]. It has been crucial for pharmacists to address patient concerns on polypharmacy, medication safety and medication information via telehealth and telephone counselling [3,11,14,16,17,18].

A useful way to provide medication adherence support and improve patient literacy has been by supplementing counselling with written information in the form of medicines lists [14,19,20,21,22]. Personalized medicines list is a necessary tool for all patients, particularly those with multiple chronic conditions. There are a number of reasons for this. Medicines lists function as a memory aid for patients to remember complex dosing schedules and understand the purpose and correct way to administer a medication; they are also a useful resource for patients to support self-management of their medications [22,23,24]. Studies have shown that the provision of medicines lists to patients with low health literacy resulted in significantly improved medication adherence, perceived knowledge of medications, medication self-efficacy and patients’ responsibility in their health journey [25,26,27]. Medicines lists are also important to maintain patient awareness of their medication regimen so they can keep track of any medication supply changes from disruptions due to drug shortages, exemptions to usual prescription quantities to accommodate social distancing to reduce risk of contracting COVID-19, or new supply restrictions to ensure equitable access to certain medications during the pandemic [28,29]. In these times, updated medicines lists are a crucial source of information containing useful details of medications and allergies for healthcare professionals, family members and caregivers of patients who may fall ill suddenly and unexpectedly [24,30].

Pharmacy students are future healthcare professionals who will largely take on patient-facing roles in the hopes of optimizing patient health outcomes by improving patient literacy to their medications, providing educational health information and supporting patients in self-management of their medication regimen [8,12,31,32]. It is imperative that pharmacy students receive adequate training to build effective communication skills and develop health-literacy competencies in their undergraduate curriculum [31,33,34]. Recent research indicates that training undergraduate healthcare students in health-literacy competencies enhances their ability to communicate information in a coherent and clear manner, contributing to effective and meaningful consultations that lead to greater patient empowerment [31,35,36,37,38]. This emphasizes the importance of familiarizing pharmacy students to the benefits of medicines lists. It also underscores the need to train pharmacy students sufficiently in educating patients about their medication regimen, using a medicines list. 

Performance-based assessments are widely used in pharmacy education, to assess the skill development of pharmacy students in their clinical knowledge, communication skills and empathy. These assessments are often used as an efficient strategy or best-practice method in assessing pharmacy students’ ability to conduct a set task. The use of simulation in teaching is an effective form of performance-based assessment and commonly used in pharmacy education to assess a variety of clinical and non-clinical skills by replicating scenarios, using simulated patients and mirroring real-life clinical situations or settings [39]. These scenarios are adapted, in varying difficulty, to the level suited to the pharmacy students as they progress through their pharmacy education [39]. This allows pharmacy students to maximize learning opportunities, to practice in a safe environment and to be adequately trained for the eventuality of patient contact in real practice.

Studies have shown that a multifaceted active-learning approach using interactive teaching and learning in the form of lectures, workshops, pharmacist–patient video vignettes, and simulation-based training and assessments are useful in supporting health education for pharmacy students [39,40]. In particular, these approaches have been shown to enhance student knowledge and confidence to communicate effectively when providing health information to patients with low health literacy [39,40]. Therefore, using simulation in pharmacy health-literacy education is an important education method of assessing a pharmacy student’s ability to improve awareness of patient literacy to using medicines lists for medication self-management. 

## 2. Simulation in Pharmacy Health Literacy Education

In the second year of the pharmacy course at Monash University, pharmacy students are introduced to the topic of health literacy. Their learning outcomes include describing the importance of health literacy in healthcare, describing the attributes of a health literate organization, and applying and demonstrating key universal precautions principles. The health-literacy topics were covered over a two-week period, with a consistent curricular approach agreed in the course during COVID-19 (Table 1) [41].

In the pharmacy course at Monash University, students are assessed formatively and summatively on various skills ranging from problem solving and critical thinking to oral communication in performance-based assessments, at every year level, in Objective Structured Clinical Examinations (OSCEs) or role-play activities. The assessments simulate a real-life practice in which an individual student is appraised based on objective evaluation through direct observation [42]. Unlike conventional written examinations, performance-based assessments allow the examiners to evaluate critical soft skills in students, particularly in communication, as well as people skills. 

The second-year pharmacy students at Monash are assessed on the health literacy topics in both the conventional written examination (at the end of the semester) and performance-based assessment (at the completion of the health literacy topics). In the performance-based assessment, students are assessed on their communication skills and application of universal precautions to communicate to a patient on what a medicines list is. The students are also assessed on the importance of a medicines list, including how to use and keep one in the context of health literacy, all of which they are exposed to in the two weeks of didactic materials (Table 1). In this mode of assessment, the student will role-play the pharmacist-in-training. The student is given 7 min to interact with a simulated patient in an OSCE-style activity. The examiner, who plays a dual role, normally simulates the patient. The performance of the students is graded based on a standard marking rubric that is clearly structured with criteria that assess their communicative effectiveness and application of interpersonal communication to educate patients on the medicines list. Each criterion is predefined by performance indicators that ensure objectivity in the grading process. Students are also exposed to and assessed formatively in workshops during the two weeks. 

## 3. Challenges Faced due to COVID-19

As the world came to a standstill with the COVID-19 pandemic, the education system around the globe swiftly shifted gears to remote online learning. Teaching and learning activities had to be undertaken remotely, with the additional use of digital platforms such as Zoom^TM^, Skype^TM^, Google Meet^TM^ and Microsoft Teams^TM^ to meet educational learning outcomes. While this rapid adaptation has allowed for reasonable continuity of educational activities, it is not without its own set of challenges, especially in areas where learning outcomes may be difficult to achieve with online learning. For example, performance-based assessments are onerous to simulate online, as they are high-stakes and time-sensitive. Regardless, many pharmacy educators globally have been utilizing video conferencing tools such as Zoom^TM^ to assess OSCEs and role-play activities synchronously online [43].

One of the objectives of the medicines list role-play assessment in the second-year pharmacy course is to appraise the students’ communication competency, which includes both verbal and non-verbal skills. An effective communication, according to the Mehrabian communication model follows a 55/38/7 rule, whereby 55% comprises non-verbal cues such as body language, 38% vocal tone and 7% spoken words [44]. As non-verbal cues account for a substantial weightage in an effective communication process, a thorough assessment of this component is integral to the overall appraisal of the students’ communication skills. However, on video-conferencing platforms, it can prove to be challenging to capture the full range of students’ non-verbal responses, due to various technical limitations, such as screen size, camera position and poor resolution [43,45].

In addition, equity issues surrounding internet access and connectivity amongst students also play a major role that may impede objective assessment of synchronous online role-play type activities. Students living in rural and remote areas where internet access can be sparse, as well as students who are unable to afford sufficient internet bandwidth, can find tasks such as these challenging. Low bandwidth connection may severely affect their internet speed leading to lagging and disjointed interactive communication. As such, assessment of time-sensitive activities can become problematic on digital platforms.

The duality of the Monash Pharmacy course adds another dimension of complexity for these assessments, as it is taught across two countries (Malaysia and Australia) synchronously. Due to this, clear instructions on assessment tasks and criteria had to be provided consistently across students in both countries, during the rapid shift to online delivery. It was also equitably essential to ensure that students from both campuses had adequate resources to undertake the online assessments fairly and uniformly. Despite these challenges, with clear and regular communication between instructors in both countries, the teaching and learning activities were carried out seamlessly during this unexpected global crisis.

## 4. Adapting to Remote Learning during COVID-19

### 4.1. Strategies to Ensure Equity and Feasibility

With the considerable challenges of synchronous virtual role-play assessment in mind, adaptations were made to the existing role-play assessment, which was originally designed to be conducted face-to-face. In adapting an alternative mode of assessment, educators were cognizant that it was imperative for the new design to remain strongly aligned with the learning outcomes of the topic. At the same time, the assessment also had to be technically feasible for students to undertake remotely. Thus, the concept of a video-recording assessment was conceived.

Students were required to submit a video recording of themselves educating a simulated patient on a medicines list. As the task was essentially carried out under lockdown restrictions during the COVID-19 pandemic, students performed the role-play with simulated patients, consisting of close contacts, such as their family members, housemates or peers at home, either in person or via video conferencing on Zoom^TM^. This meant that students were able to carry out this task in their own time, without the pressures of relying on the synchronous use of internet access. The students were also able to decide how best to undertake the assessment based on the choice of technology that was available to them, with respect to their individual circumstance.

### 4.2. Clear Instructional Material

Educators ensured that students were supplemented with very clear guidelines and instructions for the assessment (Table 2). These included the following:

- An overview of the assessment and details, including the specific format of the role-play, where the students need to educate the simulated patient on what is a medicines list, the importance of a medicines list, and how to keep a medicines list, as well as demonstrate teach-back and elicit questions from the patient that they may have about their medicines.
- Written instructions on what options are available to record the role-play (e.g., smartphone and video-conferencing tools); how to record the role-play (e.g., step-by-step instructions and time limit); and when, where and how to submit their recordings (e.g., instructions on how to compress files and submit for grading).
- Assessment criteria, including marking rubrics that the student videos will be graded on.
- A sample video recording of the required task, for their reference.
- A video recording of the instructor guiding students through the assessment instructions (in addition to the written instructions, as stipulated above).

The students were provided with sample recordings of the required task, as stated above. They would then advise the simulated patient on what was required of them in the role-play. The simulated patient’s role was of a limited nature in this task. They had to receive education on what a medicines list is, theimportance of a medicines list and how a medicines list should be kept.. The simulated patient was then required to demonstrate teach-back of the information acquired. Inaccuracies in teach-back would be reflective of the student’s ability to provide effective communication to the simulated patient. The student would also be required to elicit questions from the simulated patient about their medicines, as this task was created to assess student preparation to undertake this activity on experiential placements under the supervision of a pharmacist. As such students were trained only to elicit questions and direct them to the supervising pharmacist. They were not permitted to answer these questions. The student’s performance was not affected by the questions asked by the simulated patient. Overall, the student’s performance was not dependent on the simulated patient, and therefore formal training of simulated patients was not required.

Due to the easy accessibility to edit videos, it was also important to highlight to students that editing of videos was not allowed and only one-take recordings were accepted. With the advent of recording features in all handheld devices and the current generation of students being digitally savvy, the execution of this task was easily accomplished by all the students, with recording submissions received from a total of 304 students. 

### 4.3. Preparation before Assessment

The students were provided with many opportunities to develop health-literacy skills and practice their role-plays in a formative environment. This occurred either during interactive lectures or as part of the workshop activities. Students were provided with examples of pharmacist–patient role-plays during the interactive lectures and were required to identify and suggest possible ways the pharmacist could have improved the patients’ health-literacy awareness, such as incorporating the teach-back technique or encouraging the patient to ask questions. Feedback videos were also provided to the students, so they could learn the importance of these steps in increasing health-literacy awareness during patient-education sessions. These concepts were reinforced further by showing a different set of role-play video examples to the students during the workshops, to better prepare the students for the health-literacy assessment task. The workshop activities were also adapted to provide additional time for students to work in small groups via breakout rooms over the Zoom^TM^ meeting, so they could practice familiarizing themselves with doing simulated pharmacist—patient role-plays. Students had access to the assessment marking rubric, which consisted of seven criteria, covering a range of communication and interpersonal skills, with clearly outlined, predefined performance indicators. The students utilized this rubric early on by providing peer feedback in the form of the Keep-Start-Stop model, where a student would receive feedback on what they should “keep” doing or what was done well, what they should “start” doing or could be improved upon, and what was ineffective which they should “stop” doing.

### 4.4. Grading of Assessment

Examiners who are practicing pharmacists graded the videos. Examiners were given a marking rubric, a feedback template that uses the Keep–Start–Stop framework. In addition, examiners were initially requested to grade the first 5 students. The academic lead for the topic graded the same 5 students independently. Grades were compared and any discrepancies were discussed to ensure consistency and standardization in marking. This initial moderation was conducted amongst graders in the local campus as well as across both Malaysian and Australian campuses, to ensure inter-campus grading consistency.

Each role-play is a maximum of 7 min. Similar to face-to-face role-plays, the examiners were given 7 min with the students and an additional 2 min to complete marking. In total, about 46 h were allocated to grade 304 submissions across both campuses. We had a number of examiners, and each examiner had a smaller number of students to grade. On average, each examiner took approximately 5 h to complete 35 video submissions. When all the videos were graded, the academic lead carried out a final review of the grading and examiner feedback, to ensure all students received a fair assessment. Consistency and standardization in grading were ensured through the moderation processes.

## 5. Lessons Learned

There have been concerns about maintaining academic integrity over an online platform that can be difficult to achieve across the video-conferencing tools that are presently available. Collusion and cheating among students are external factors that are difficult to control and may jeopardize the integrity of these assessments [45]. However, this particular performance-based assessment has a core focus on communicative effectiveness, without a problem-solving component, that makes collusion and cheating irrelevant.

Some instructors were apprehensive with the adaptations to the assessment, as they felt that students would only submit their “best” video recording. However, studies have shown that using deliberate practice can improve communication skills [47]. If students are repeating the task numerous times to perfect their assessment submission, they will improve their communication skills through repeated practice, while indirectly enhancing their self-confidence.

In addition, many of the students included their close contacts, such as family members and housemates, to roleplay as the simulated patient in their video recordings, due to isolation requirements faced during the COVID-19 pandemic related lockdown restrictions. Through this role-play exercise, these are members of the public who are now educated on the importance and use of a medicines list who may not have been exposed to the concept of medication self-management by using this important health-literacy tool prior to this. That is one additional person who is now aware of and knows how to keep an accurate medicines list that is even more crucial during uncertain times, such as the COVID-19 pandemic.

Furthermore, there were also parents of pharmacy students simulating the role of patients in their children’s video recordings. This would have served as a great opportunity for them to see what their children are capable of as part of their education as pharmacists-in-training.

Moving forward, role-play video recordings may be adopted into the health-literacy curriculum, as a small component of assessment, given its feasibility and hidden advantages. Structured evaluation of this mode of assessment is warranted to objectively quantify the outcome of this exercise.

## 6. Conclusions

The COVID-19 pandemic has challenged the traditional framework of our education system, spurring educators around the world to make systematic yet strategic innovations in their teaching and learning methods. Pharmacy education in particular, has witnessed a remarkable shift during this unprecedented time, creating meaningful and relevant opportunities for students to prepare themselves for the imminent digitalization of healthcare. Introducing students to simulation activities via telehealth, to increase health literacy, is clearly a silver lining in this extraordinary circumstance. The collaborative effort of pharmacy instructors and pharmacy students with members of the public indirectly increased the awareness of patient literacy to utilizing medicines lists for medication self-management through this task. Simulated role-play activities that involve pharmacy students and members of the public is a viable option to be incorporated into the pharmacy course.

## Figures and Tables

**Table 1:** Curricular approach to health-literacy content [41].

Curricular Element	Approach to Content	Learning Objectives
Pre-class online learning material	Activities include assigned articles, videos and recordings that match the learning outcomes	Define health literacy. Articulate why health literacy is important in healthcare. Appreciate the extent of the problem of limited health literacy globally and the costs to health care systems. Describe the impact of limited health literacy on patients and their utilization of health care services and medicines. Describe what universal precautions are and why they are an appropriate set of communication principles in this context.
Interactive lectures	An interactive lecture built into the learning management system (i.e., Moodle) for asynchronous delivery. Lecture consists of clips of video segments, followed by questions and activities including instructor feedback	Observe for clues (“red flags”) to limited health literacy in patients. Reflect on the impacts of limited health literacy and devise recommendations for professionals when interacting with someone who has limited health literacy. Create conceptual links between responses to a scenario.
Workshops	Live-streamed workshops through video-conferencing technology (i.e., Zoom^TM^ Meeting) for synchronous delivery. The “breakout room” feature of Zoom^TM^ is used to allow for two to three students in a room for role-plays where they simulate the medicines list counselling education	Apply universal precautions to interactions with patients. Demonstrate two key universal precautions principles—teach-back and asking “What questions do you have?”—in response to given scenarios in interactive lectures, workshops and the assessment task, in preparation for patient interaction on placement. Elicit questions that patients have about their medicines in response to given scenarios in the workshops and the assessment task, in preparation for patient interaction on placement.
Close-the-loop sessions	Audience response questions (i.e., Poll Everywhere) embedded into learning management system (i.e., Moodle) at the end of each week for students to ask questions about the topic. The session is then live-streamed through video-conferencing technology (i.e., Zoom^TM^ Webinar) where instructors respond to questions with synchronous delivery.	List questions about content that require clarification.

**Table 2:** Instructions provided to students for the performance-based assessment [46].

Instructional Material
Overview:
The assessment is a performance-based assessment of your communication skills and application of universal precautions to communicate with a simulated patient. You are required to submit a role-play video for this Health Literacy OSCE. This assessment is a role-play with a simulated patient (of your choice) where you will be assessed on performing three points of identification, applying universal precautions to communicate what is a medicines list, why it is important to a patient, and how to use and keep it. You will also elicit questions the patient has for their pharmacist and demonstrate teach-back.
Video recording of role-play:
An example video of the required activity is available here http://bit.ly/HealthLitVid. Please select another individual (e.g., another student, family member or friend) to educate them on a medicines list. You need to play the role of the pharmacist-in-training for your scenario where you educate another individual on a medicines list. You may choose to record this in your own home with your own recording device (e.g., phone). If using your phone to record, please ensure your phone is in landscape orientation, to make viewing easier. If you are in self-isolation on your own, you may pair up with another individual and complete this task and recording via Zoom^TM^. Video record the interaction (ensure both patient and pharmacist are within frame). The maximum length of the role-play should be 7 min.
Specific format of the task includes the following:
Professional attire is required in your role-play video. You will complete only one role-play video. As part of your task, you will need to do the following: ◯ Perform three points of identification ◯ Educate the simulated patient on what a medicines list is ◯ Educate the simulated patient on the importance of a medicines list ◯ Educate the simulated patient on how to keep a medicines list ◯ Demonstrate teach-back ◯ Elicit questions from the patient that they have about their medicines.
